# Transjejunal Laparoscopic Assisted ERCP in a Patient with Roux-en-Y Hepaticojejunostomy

**DOI:** 10.3390/medicina55080483

**Published:** 2019-08-14

**Authors:** Adrian A. Baca-Arzaga, Hector Navarro-Chavez, Jesus Galindo-Jimenez, Jose Santibanez-Juarez, Claudia Cardosa-Gonzalez, Eduardo Flores-Villalba

**Affiliations:** 1 Escuela Nacional de Medicina, Tecnologico de Monterrey, Monterrey 64710, Mexico; 2 Hospital Regional ISSSTE Monterrey, Monterrey 66603, Mexico; 3 Escuela Nacional de Ingeniería, Tecnologico de Monterrey, Monterrey 64849, Mexico

**Keywords:** Roux-en-Y hepaticojejunostomy, hepaticojejunal anastomosis, endoscopic retrograde cholangiopancreatography, hepatic cholelithiasis, biliary injury repair

## Abstract

*Background and Objectives:* Nowadays, with the increasing laparoscopic expertise and accessibility to modern surgical tools, laparoscopic assisted ERCP (LAERCP) has become an effective approach for the management of bile stone disease in patients with modified gastrointestinal anatomy. In contrast to patients with gastric bypass in whom a transgastric LAERCP approach is usually performed, the resultant anatomy of Roux-en-Y hepaticojejunostomy precludes a gastric approach as the newly formed bilioenteric anastomosis is not reachable through the stomach. Therefore, a transjejunal approach has been described as an alternative LAERCP technique. To the best of our knowledge this is the tenth case of transjejunal LAERCP reported worldwide. *Materials and Methods:* We present the case of a 50-year-old female with history of biliary injury during a cholecystectomy corrected with Roux-en-Y hepaticojejunostomy who presented to our center with manifestations of acute abdomen. After laboratory and image analysis, diagnosis of intrahepatic lithiasis was confirmed. The decision to perform a transjejunal LAERCP was made due to the complex anatomy in this patient. No complications were found during surgery and in the follow up period. *Conclusions:* Transjejunal LAERCP is an effective approach for endoscopic management of biliary complications in patients with Roux-en-Y hepaticojejunostomy and other modified gastrointestinal anatomy. Previous recommendations by more experienced teams have been reported, nonetheless, there are too few cases reported to make definitive recommendations and conclusions. In limited settings, such as ours, some of these recommendations may not be applicable. We are certain that, with the increasing expertise and innovations in laparoscopy surgery for the management of complications that cannot be addressed by endoscopic or noninvasive measures, more cases will be reported.

## 1. Introduction

Management of complications in patients with surgically modified gastrointestinal anatomy is still today an important surgical and endoscopic challenge. Among its many indications, Roux-en-Y hepaticojejunostomy (RYHJ) has been described as the preferred approach for biliary tract continuity alterations, either by primary or secondary causes [1]. Despite its positive outcomes, modified digestive tract anatomy after RYHJ renders future biliary endoscopic instrumentation non-viable as the length from the oral cavity to the bilioenteric anastomosis is increased.

Laparoscopic assisted endoscopic retrograde cholangiopancreatography (LAERCP) has been recently described as an effective alternative for modified anatomy, especially in patients with Roux-en-Y gastric bypass (RYGB) with a reported overall success rate of 98% [2]. Nonetheless, in patients with RYHJ a transjejunal approach has been described as an alternative to the transgastric approach used in RYGB anatomy. Although experience is minimal with few cases reported to date, it has shown promising results [3,4,5,6,7,8,9,10].

The objective of this report is to present a case of transjejunal LAERCP in a patient with surgically modified gastrointestinal anatomy, which to the best of our knowledge, is the tenth case reported worldwide.

## 2. Case Presentation

**Figure 1:**
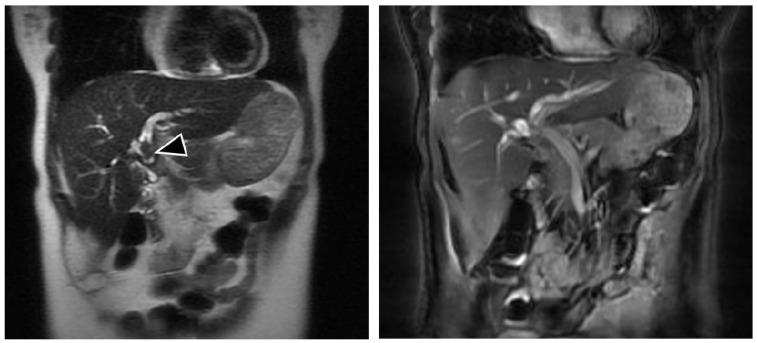
Magnetic resonance cholangiography showing the filling defect in the hepatic bile duct (arrow).

We report a case of a 50-year-old overall healthy female with history of biliary injury during cholecystectomy repaired with RYHJ 25 years ago as well as open hepatic adenoma resection and splenectomy 4 years ago. Her follow-up was uneventful until 2 years ago when she started with episodes of abdominal pain localized in the right upper quadrant, associated with fever and non-bilious emesis, which were managed by her local physician with analgesics and antibiotics. In the previous days, the pain had dramatically intensified and she was referred to our center for surgical evaluation. Physical examination revealed abdominal pain on deep palpation in the lower right hypochondrium, with negative peritoneal signs. Vital signs were all in normal ranges for her age. Laboratory analyses were as follows: leukocytes 13.9 cells/mm^3^; neutrophils 8.6 cells/mm^3^; total bilirubin: 0.7 mg/dL; alanine transaminase: 173 U/L; aspartate transaminase: 130 U/L; alkaline phosphatase: 1162 U/L, GGT 300 U/L, amylase: 300 U/L, and negative viral hepatitis panel. Magnetic resonance cholangiography (MRC) revealed intrahepatic duct dilation and lithiasis (Figure 1).

Owing to the history of RYHJ, it was decided to perform a transjejunal LAERCP under general anesthesia using the standard laparoscopic approach with four trocars.

**Figure 2:**
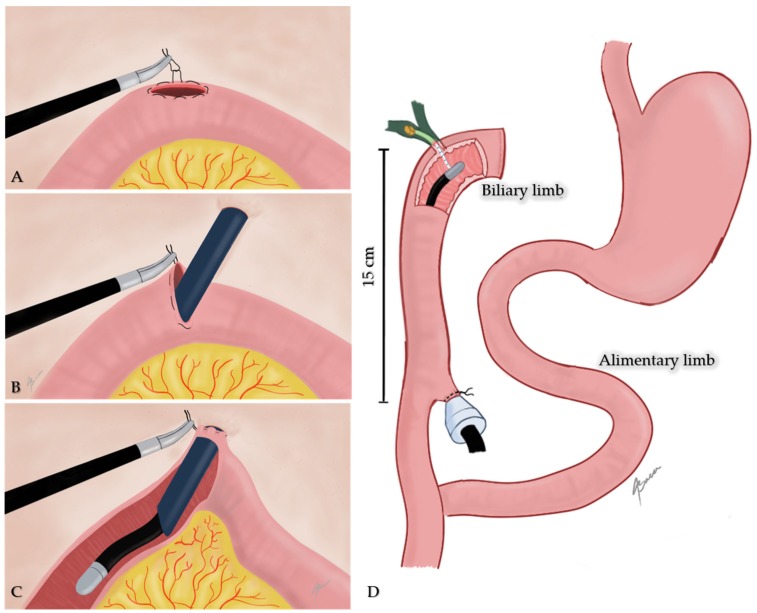
Illustration showing the transjejunal approach procedure. (**A**) Purse-string suture around enterotomy; (**B**) trocar insertion through enterotomy; (**C**) intestinal limb pulled in close contact to abdominal wall and purse-string tightening; (**D**) endoscope insertion through biliary limb.

An initial endoscopical exploration revealed no alterations in the jejunojejunal anastomosis. Once the biliopancreatic limb was identified, a 15 mm enterotomy 15 cm distal to the hepaticojejunal anastomosis was performed. A purse-string with a silk stitch was sutured around the ostomy. Thereafter, a 15-mm trocar was introduced through the umbilical access-port into the jejunum; the latter was pulled in close contact to the abdominal wall and secured by tightening the purse-string stitch (Figure 2). This was in order to avoid leaking of intestinal content and facilitate endoscope maneuvering.

**Figure 3:**
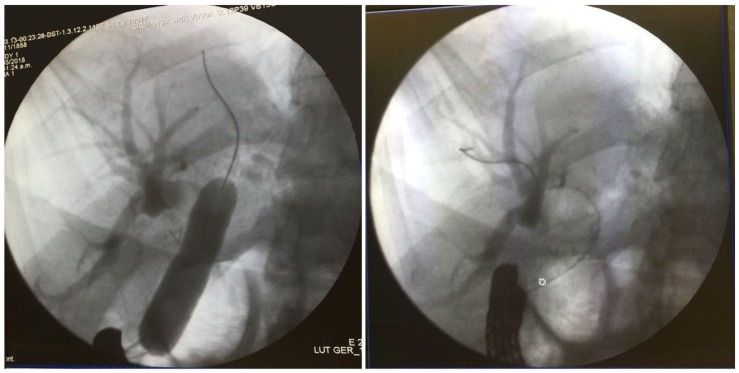
ERCP showing balloon dilation of bilioenteric anastomosis and intrahepatic biliary ducts.

**Figure 4:**
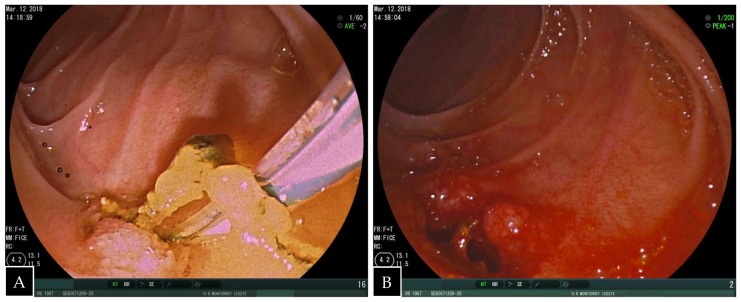
(**A**) Endoscopic view of the duodenum showing retrieval of biliary sludge and stones and (**B**) dilated bilioenteric anastomosis.

A standard side-viewing endoscope (Olympus TJF 160 VR or TJF 145, Tokyo, Japan) was advanced through the jejunal trocar in caudocephalad direction until the papilla was visualized. Once successful catheterization of the papilla was obtained, ERCP was carried out in a standard fashion showing a filling defect in the common hepatic duct proximal to the anastomosis. Balloon dilation of the anastomosis was required (Figure 3). A single 5 mm stone and biliary sludge were removed with a retrieval balloon catheter. Subsequent cholangiography showed no evidence of filling defect. The enterotomy was closed with continuous sutures in double layer (Figure 4). There were no incidents during the surgical procedure.

The clinical course was uneventful as demonstrated by postoperative MRC and laboratory analysis performed the day after the procedure which showed normalized liver enzymes. The patient was discharged on the third postoperative day.

## 3. Discussion

Recurrent biliary stones are described as one of the complications of RYHJ along with biliary strictures [11]. This can be explained by the direct communication between the biliary and digestive tract owing to the absence of a functional valve such as Oddi sphincter with the newly formed anastomosis. Additionally, there is an increased probability of chyme, bacteria, and air entering the biliary tract, as well as increased levels of mucin and oxygen free radicals that promote stone formation [12].

Balloon assisted enteroscopy ERCP (BAE-ERCP) is considered an alternative approach for modified gastrointestinal anatomy patients. In a recent meta-analysis that included patients with RYHJ, RYGB, and Whipple anatomy managed with single balloon enteroscopy, a relatively new BAE technique, an overall success rate of 80.9% was found [13]. Even though BAE techniques and LAERCP have not been compared among patients with RYHJ anatomy, data from patients with RYGB favors toward the laparoscopic approach [14]. Percutaneous transhepatic cholangioscopy (PTCS) is another well-described alternative. It can precisely localize the affected bile duct, and it allows repeated interventions through the percutaneous tract. Nonetheless preparation for a safe and effective PTCS may require days or even weeks. In addition, access to multiple bile ducts cannot be achieved in a single puncture. Finally, postprocedural pain requiring analgesic administration has been described as a minor but remarkable disadvantage [15].

Nine cases of transjejunal LAERCP have been reported since 2007 (Table 1). Though some cases include patients with previous RYGB, in which a transgastric approach might seem a better option, the justification mentioned in these cases supports the decision in favor of a transjejunal approach. As an example, in the Surdeanu et al. case, severe adhesions found during laparoscopy precluded a transgastric approach. In other cases, a conventional trans oral approach ERCP was attempted which was unsuccessful as the endoscope could not reach the biliary papilla [7,8,10]. All of these cases had RYGB anatomy, whereby, in contrast to our patient, there is an intact biliary papilla, and direct access from the gastric pouch is anatomically viable.

A single case in a young patient with RYHJ has been reported by Mansor et al., who in contrast to our case, performed bowel exteriorization through the optic port incision as part of their technique for endoscopic cannulation [10]. Two authors have recently addressed the importance of this maneuver to prevent intestinal content spilling into the abdominal cavity during enterotomy creation and to facilitate bowel cannulation, though in their cases it was performed through a mini-laparotomy [3,4].

As we did not experience difficulties during bowel cannulation and patient recovery did not evidence infectious complications, in our opinion a mini laparotomy can add morbidity to the surgery and might be unnecessary as the socket technique first described by Mutignani in a transjejunal approach has been used also in transgastric LAERCP with excellent results [2,9].

Endoscope selection is also an important discrepancy among the reported cases. Only two cases have explained their preference towards using a colonoscope in their techniques, which state it has a reduced risk of intestinal injuries due to the limited visual field of side viewing with other endoscopes [3,4]. In our case, side viewing was not an issue, and it even permitted us to localize the bilioenteric anastomosis and provided us the stability for an adequate biliary cannulation. Nonetheless, we agree with other authors that in cases with native papilla, an inverted view secondary to anterograde approach can be a considerable struggle; therefore, we support the recommendation that, if available, a frontal view endoscope might be a better option.

## 4. Conclusions

Transjejunal LAERCP is an effective approach for endoscopic management of biliary complications in patients with Roux-en-Y hepaticojejunostomy and other modified gastrointestinal anatomy. This case demonstrates that, although previous recommendations by more experienced teams are important for optimal results, in limited settings such as in our case, some of these recommendations may not be applicable. Still, there are too few cases reported to make definitive recommendations and conclusions; however, we are certain that, with the increasing expertise and innovations in laparoscopy surgery for the management of complications that cannot be addressed by endoscopic or noninvasive measures, more cases will be reported.

## Figures and Tables

**Table 1:** Transjejunal LAERCP reported cases.

Author	Age	Gender	GI Surgery	Bypass Time (months)	Diagnostic Studies	Diagnosis	LOS (Days)	Follow up	Concomitant CCE	Endoscope Type	Notes
Dalmonte 2019	NM	Female	RYGBP	24	CT, MRCP	CBD stones	4	3m	yes	Colonoscope	Biliary limb extracted through mini laparotomy
Mita 2018	79	Female	RYGBP	48	CT, MRCP	CBD stones, CCL	6	NM	yes	Colonoscope	Biliary limb extracted through mini laparotomy
Marchesini 2017	60	Male	BPD	24	MRCP	CBD stones, CCL	NM	NM	yes	Duodenoscope	Rendezvous cannulation through gallbladder, bowel drawn up to abdominal wall.
Marchesini 2017	52	Female	BPD	36	US, MRCP	CBD stones, CCL	NM	NM	yes	Duodenoscope	
Surdeanu 2016	50	Male	RYGBP	48	US, IOC	CBD stones, CCL	7	NM	yes	Endoscope (?)	Jejuno-jejunal anastomosis exteriorization
Mansor 2015	17	Female	Roux-en-Y hepaticojejunostomy	156	MRCP	Ascending cholangitis due to intrahepatic gallstones	5	12m	No (previously done)	Gastroscope	Bowel drawn up to abdominal wall through optic port incision
Lopes 2010	18	Female	RYGB (partial gastrectomy)	24	CT, MRCP	Sphincter of Oddi dysfunction	NM	3m	No (previously done)	Duodenoscope	No exteriorization
Saleem 2010	47	female	ST gastrectomy with RY gastrojejunostomy	NM	NM	Pancreaticopleural fistula	5	2m	NM	Duodenoscope	Biliopancreatic limb drawn up to abdominal wall through right subcostal port

CCE: cholecystectomy; CCL: cholecystolithiasis; CBD: common bile duct; IOC: intraoperative cholangiogram; LOS: length of stay; MRCP: magnetic resonance cholangiopancreatography; NM: not mentioned; RYGBP: Roux-en-Y gastric bypass.
